# rhHMGB1 drives osteoblast migration in a TLR2/TLR4- and NF-κB-dependent manner

**DOI:** 10.1042/BSR20150239

**Published:** 2016-02-19

**Authors:** Ming-Jing Li, Fan Li, Jian Xu, Yu-Dong Liu, Tao Hu, Jian-Ting Chen

**Affiliations:** * Department of Orthopedic and Spinal Surgery, Nanfang Hospital, Southern Medical University, Guangzhou 510515, China; † Department of Pediatric Orthopedic, Wuhan PuAi Hospital, Tongji Medical College, Huazhong Science and Technology University, Wuhan 430030, China

**Keywords:** migration, osteoblast, proliferation, recombinant human high mobility group box 1 protein (rhHMGB1), siRNA, skeletal development

## Abstract

Osteoblast migration is significant in skeletal development. Recently, high mobility group box 1 protein (HMGB1) has been shown to highly expressed in cartilage to regulate endochondral ossification. Nevertheless, whether HMGB1 can modulate osteoblast proliferation and migration is poorly understood, as well as the intracellular signalling pathways that are involved in this process. Herein, we examined the effects of recombinant human HMGB1 (rhHMGB1) on the proliferation and migration of rat osteoblasts and investigated whether Toll-like receptor 2 (TLR2)- and TLR4-dependent signalling pathways are involved in the regulation of intracellular signalling. A transwell chamber assay was used to evaluate the migration of osteoblasts and the MTT assay was used to assess osteoblast proliferation. rhHMGB1 could significantly promote the migration of osteoblasts without inhibiting their proliferation. Meanwhile, rhHMGB1 can increase the nuclear translocation of nuclear factor-kappa B (NF-κB) p65. Specific siRNA constructs that target *TLR2* or *TLR4* could markedly inhibit HMGB1-induced migration of osteoblasts and HMGB1-enhanced activation of NF-κB. Collectively, HMGB1 could significantly enhance the migration of osteoblasts *in vitro*, and TLR2/TLR4-dependent NF-κB pathways are involved in HMGB1-induced osteoblast migration.

## Abstract (precis)

In this paper, we reported that rhHMGB1 could significantly enhance the migration of osteoblast without causing cytotoxic effects through the activation of NF-κB via TLR2 or TLR4, indicating a significant functional role for HMGB1 in skeletal development and bone restoration.

## INTRODUCTION

The proliferation and migration of osteoblasts is important for both skeletal development and bone fracture healing [1]. Previous studies have shown that many extracellular cytokines, such as bone morphogenetic proteins (BMPs), insulin-like growth factors (IGFs) and wingless and int (Wnt) ligands are involved in the proliferation and migration of bone cells [2–4]. High mobility group box 1 protein (HMGB1) is a non-histone nuclear protein that is expressed in all eukaryotic cells [5]. HMGB1 can be passively released by necrotic or damaged cells [6] and actively secreted by activated monocytes or macrophages [7]. Since it was initially reported as a lethal mediator of sepsis by Wang et al. [8], extracellular HMGB1 has been reported to act as a novel cytokine that contributes to inflammation [9–11], reperfusion after ischemia of skeletal muscle tissue [12], liver fibrosis [13] and tumour growth and metastases [14,15].

Recombinant human HMGB1 (rhHMGB1) can promote the proliferation and migration of myofibroblasts, skeletal myoblasts and mesoangioblasts [16–18]. Previous reports have shown that rhHMGB1 is a factor that can exert bone bioactivity to induce osteoclast formation and differentiation of bone marrow stem cells to osteoblasts [19]. rhHMGB1 can be released from bone cells, including osteoblasts and osteoclasts, as well as MLO-Y4 osteocyte-like and MC3T3-E1 osteoblast-like cells [20–22]. A previous study reported that hypertrophic chondrocytes within growth plates could release HMGB1 to regulate endochondral ossification. Analyses of *Hmgb1*^−/−^ embryos indicated that long bone development is significantly compromised by HMGB1 deficiency [23]. These findings suggest that extracellular HMGB1 may contribute to embryo skeletal development. We propose that in skeletal development during embryogenesis, the release of modest amounts of HMGB1 can regulate the number of osteoblasts and osteoclasts that are recruited to the breach in the ossification centre. However, the role of HMGB1 in bone and cartilage tissues that undergo remodelling during embryogenesis remains less clear.

Extracellular HMGB1 is a ligand for Toll-like receptor 2 (TLR2), TLR4 and the receptor for advanced glycation end products (RAGE) [12,24–26]; osteoblasts express all of these known receptors for HMGB1 [22]. A previous study showed that extracellular HMGB1 could promote the migration of human dermal microvascular endothelial cells via TLR4 [12]. However, the biological function of the HMGB1–TLR axis in osteoblasts remains incompletely understood. Based on the aforementioned earlier reports, our present study aimed to investigate the potential role and regulatory mechanisms of rhHMGB1 in the proliferation and migration of rat osteoblasts. Overall, our data showed that rhHMGB1 could significantly promote the migration of osteoblasts without causing cytotoxic effects through the activation of nuclear factor-kappa B (NF-κB) via TLR2/TLR4, indicating the potential utility of targeting HMGB1 for the treatment of severe bone-related diseases.

## MATERIALS AND METHODS

### Materials

Osteoblasts from SD rats were purchased from Weikai Bioeng. rhHMGB1 was obtained from Sigma. FBS, dulbecco's modified eagle media (DMEM), alpha minimal essential media (α-MEM) and antibiotics (100 U/ml penicillin and 100 μg/ml streptomycin) were purchased from Invitrogen. Antibodies against HMGB1, TLR2, TLR4, NF-κB p65 and β-actin were purchased from Abcam. NE-PER nuclear and cytoplasmic extraction kit (Pierce).

### Cell culture

Osteoblasts were cultured in DMEM medium, supplemented with 10% FBS, 100 U/ml penicillin and 100 μg/ml streptomycin and incubated at 37°C with 5% CO_2_; medium was replaced every 2 days.

### TLR2 or TLR4 knockdown by siRNA

Following generally accepted optimization principles for siRNA design [27], we generated three sequence-specific siRNAs to target either TLR2 or TLR4. Additionally, a negative control siRNA (NC-siRNA) that showed no homology with the human genome was designed as a negative control. The siRNA sequences are listed in Table 1. Osteoblasts were cultured at 37°C in a 5% CO_2_ incubator until cells were 70–80% confluent. Subsequently, on-target siRNA or NC-siRNA was transfected into cells using Turbofect siRNA transfection reagent according to the manufacturer's instructions (Thermo Scientific). After culture for 4–6 h at 37°C, serum-free DMEM was replaced with complete growth medium (DMEM with 10% FBS) and cells were cultured for an additional 48 h.

**Table 1:** Sequences of pre-designed siRNAs that targeted either TLR2 or TLR4

Name	Sequence
TLR2-siRNA 318	Forward 5′-GCGGAAUCAACACAAUAGATT-3′
	Reverse 5′-UCUAUUGUGUUGAUUCCGCTT-3′
TLR2-siRNA 392	Forward 5′-CACCUAUCUAGUUUAUCUUTT-3′
	Reverse 5′-AAGAUAAACUAGAUAGGUGTT-3′
TLR2-siRNA 1647	Forward 5′-GCAGGUGACAACCAUUUCATT-3′
	Reverse 5′-UGAAAUGGUUGUCACCUGCTT-3′
TLR4-siRNA 446	Forward 5′-GCUAUAGCUUCACCAAUUUTT-3′
	Reverse 5′-AAAUUGGUGAAGCUAUAGCTT-3′
TLR4-siRNA 703	Forward 5′-GGCUCAUAAUCUUAUACAUTT-3′
	Reverse 5′-AUGUAUAAGAUUAUGAGCCTT-3′
TLR4-siRNA 2299	Forward 5′-CGAGCUGGUAAAGAAUUUATT-3′
	Reverse 5′-UAAAUUCUUUACCAGCUCGTT-3′
NC-siRNA	Forward 5′-UUCUCCGAACGUGUCACGUTT-3′
	Reverse 5′-ACGUGACACGUUCGGAGAATT-3′

### RT-PCR

The mRNA transcript levels of *TLR2* and *TLR4* were measured by quantitative real-time PCR, as described previously [28]. Total RNA was extracted from each group of cells using TRIzol (Invitrogen) according to the manufacturer's protocol. A total of 1 μg RNA was reverse-transcribed into cDNA using a RevertedAid First Strand cDNA Synthesis Kit (Fermentas). Primer sequences are listed in Table 2. Real-time PCR was performed using ABI StepOne Plus and specific primers for target genes and *Actin* (for endogenous control) in triplicate. PCR products were subjected to melting curve analysis and a standard curve that was generated to confirm amplification.

**Table 2:** Primer sequences used for RT-PCR analysis

Genes	Primer sequence
TLR2	Forward 5′-AGCTTCATTGTTCCCTGTGTTAC-3′
	Reverse 5′-AGTTCACAGGAGCAGATGAAATG-3′
TLR4	Forward 5′-CACTTTATCCAGAGCCGTTGG-3′
	Reverse 5′-GGCTACTCAGAAACTGCCATG-3′
NF-κB	Forward 5′-TATGACCTGGACGACTCTTGG-3′
	Reverse 5′-CTGTCAGCTGCTTAATGTCCC-3′
β-Actin	Forward 5′-GGGAAATCGTGCGTGACATTA-3′
	Reverse 5′-TTGCCGATAGTGATGACCTGA-3′

### Cell viability assay

The MTT method was used to assess cell viability. Cells were seeded at a concentration of 5×10^3^ cells per well in 96-well plates. After growth for 24 h, cells were treated with the indicated doses of rhHMGB1 for 24, 48 and 72 h. Cell viability was measured using the MTT (3-[4,5-dimethyl-2-thiazolyl]-2,5- diphenyl-2H-tetrazolium bromide) assay kit (Sigma) and absorbance was read using a microplate reader (Bio-Rad) at 492 nm. All experiments were carried out in triplicate.

### Cell invasion assay

Cell invasion was studied using a Boyden Transwell chamber assay (polycarbonate membrane inserts with 24 pores, pore size 8.0 μm, membrane insert diameter 6.5 mm). Cells in 100 μl serum-free DMEM media at a density of 1×10^5^ cells/ml were plated in upper chambers that were precoated with Matrigel Basement Membrane Matrix (Sigma). In the lower chamber, 500 μl DMEM medium with 15% FBS was added as a chemoattractant. After 4–5 h, Matrix gels and cells on the top membrane surface were removed using a cotton swab. Transwell membranes were stained with crystal violet, and cells were counted under a light microscope in four or five randomly selected microscopic fields (Olympus Microscope System, Olympus).

### Western blotting

Proteins from whole cell lysates were extracted using a radio-immunoprecipitation assay. Osteoblasts stimulated with rhHMGB1 were lysed in lysis buffer. Supernatants were prepared by centrifugation, electrophoresed on a 10% SDS/PAGE and blotted on to a polyvinylidene difluoride membrane. Immunoblotting was performed using antibodies specific to TLR2, TLR4 and GAPDH, followed by an horseradish peroxidase (HRP)-conjugated secondary antibody and developed using an ECL detection kit (Abcam). The relative amounts of protein bands on the blots were determined using IPP 6.0 software (Media Cybernetics Inc).

### Cell fraction and immunoblotting

Nuclear and cytosolic factions were prepared using Nuclear and Cytoplasmic Extraction Reagents kit. Lamin B1 and GAPDH were used as markers for nuclear and cytosol proteins respectively. Lysate proteins were resolved by SDS/PAGE and transferred on to nitrocellulose membranes. The membranes were incubated with TBS containing 0.1% Tween 20 and 5% skimmed milk, and then exposed to the desired primary antibodies. After treatment with anti-rabbit antibodies conjugated with HRP, the immunoreactive bands were visualized by standard ECL method.

### Statistical analyses

All experiments were performed in triplicate. Data were presented as means ± S.E.M. One-way ANOVA tests were used for statistical analyses that were conducted with SPSS version 18.0 (SPSS Inc.). To identify statistically significant differences, a threshold of *P*<0.05 was used.

## RESULTS

### rhHMGB1 promotes osteoblast migration without inhibiting cell viability

**Figure 1:**
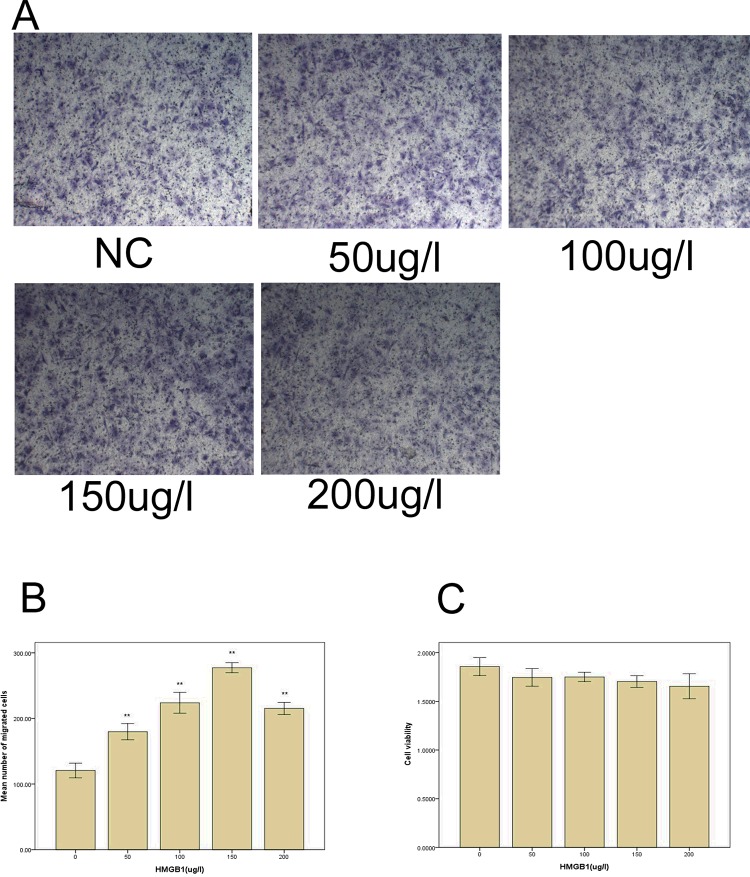
Dose-dependent promotion of osteoblast migration by rhHMGB1 without inhibiting cell viability (**A**) Osteoblasts were seeded into the upper chamber of the transwell, treated with rhHMGB1, and allowed to invade the matrigel for 4 h. The invasive cells that migrated through the basal membrane to the lower surface were stained with crystal violet, and were photographed at 10× magnification. (**B**) Migrated cells were counted under a light microscope in five randomly selected microscopic fields. One-way ANOVA was performed to determine statistical significance. rhHMGB1 promotes osteoblast migration in a dose-dependent manner (**P<0.01), which reached a peak at 150 μg/l. (**C**) Proliferation assay after incubation with rhHMGB1 for 24–72 h. Results of the MTT assay showed that rhHMGB1 had no significant effect on the cellular proliferation of osteoblasts.

To examine the effects of rhHMGB1 on the migration and viability of osteoblasts, we used a modified Boyden Chamber system and the MTT assay. We added 0, 50, 100, 150 or 200 μg/l rhHMGB1 to investigate the effects of rhHMGB1 on osteoblast activation. In a transwell chamber, many of the cells had migrated through the pores to the lower side of the membrane by 4 h, where they could be stained dark purple with crystal violet (Figure 1A). As shown in Figure 1B, our findings indicated that rhHMGB1 could promote the migration of osteoblasts in a dose-dependent manner (**P<0.01), which peaked at 150 μg/l. These data showed that the count of cells that migrated across the membrane increased 2.3-fold after 4 h of HMGB1 treatment at 150 μg/l (*P*<0.05). The MTT assay revealed that the viability of osteoblasts was not reduced significantly after treated by rhHMGB1 (Figure 1C). Therefore, 150 μg/l was chosen as an optimal concentration for use in subsequent experiments.

### Pre-designed siRNAs knockdown expression of TLR2 and TLR4

**Figure 2:**
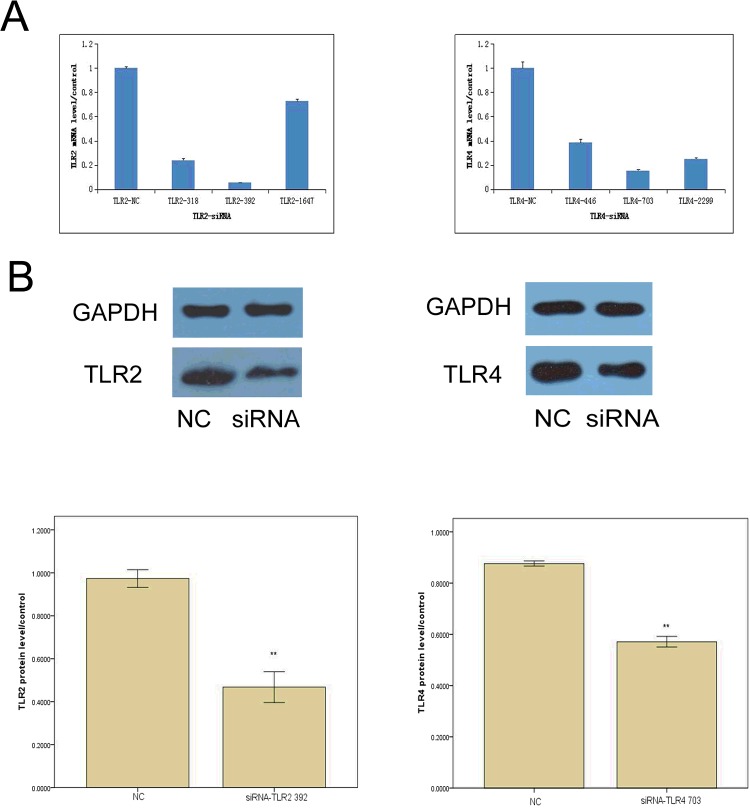
siRNA that target *TLR2* and *TLR4* reduce their mRNA transcript levels and protein levels (**A**) Knockdown of TLR2 or TLR4 mRNA transcript levels in osteoblasts by TLR2- or TLR4-siRNAs was confirmed by qRT-PCR; siRNA that target *TLR2* and *TLR4* markedly reduce their mRNA transcript levels. (**B**) Knockdown of *TLR2* or *TLR4* protein levels in osteoblasts by TLR2- or TLR4-siRNAs was confirmed by western blotting and quantified by densitometric analyses; GAPDH was used as an internal control for quantity analysis. One-way ANOVA was performed to determine statistical significance. siRNA that target *TLR2* and *TLR4* reduce their protein levels (**p<0.01).

To study the roles of TLR2 and TLR4 in osteoblasts, we examined the expression levels of TLR2 and TLR4 in osteoblasts by RT-PCR and western blotting. The mRNA transcript levels and the protein expression levels of TLR4 were higher than those of TLR2 (Figure 2A). Next, we measured the mRNA transcript levels and the protein levels of these receptors after transfection with siRNA constructs. As shown in Figures 2B and 2C, *TLR2* and *TLR4* mRNA transcript levels and protein levels were significantly reduced by specific TLR2- and TLR4-siRNA constructs. Moreover, the TLR2-siRNA_392_ and TLR4-siRNA_703_ constructs could reduce the *TLR2* and *TLR4* mRNA levels by up to 94% and 84% (*P*<0.05).

### rhHMGB1 induced NF-κB activation through TLR2 or TLR4 signalling in osteoblasts

**Figure 3:**
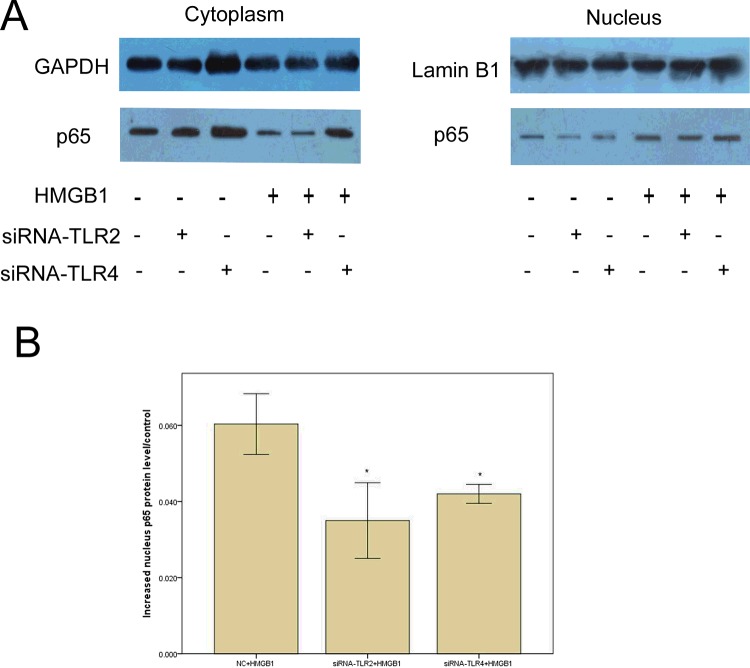
Effects of rhHMGB1 and TLR2- or TLR4-siRNA on NF-κB expression in osteoblasts (**A**) Osteoblasts were pretreated with TLR2- or TLR4-siRNA for 24 h, followed by the addition of rhHMGB1 (150 μg/l) to the culture medium for 24 h. Cells were harvested and fractionated into the cytoplasm and the nucleus. Lysates were then separated on a 10% SDS/PAGE and subjected to western blotting with anti-p65. GAPDH and Lamin B1 were used as markers for the cytoplasmic and nuclear fractions. (**B**) The NF-κB p65 protein levels that were translocated to the nucleus were quantified by densitometric analyses. One-way ANOVA was performed to determine statistical significance. Pretreatment with TLR2- or TLR4-siRNA decreased nuclear NF-κB p65 subunit levels, whereas rhHMGB1 enhanced it (*P<0.05).

To investigate the potential mechanisms whereby HMGB1 can regulate osteoblast migration, we incubated rat osteoblasts with rhHMGB1 at a concentration of 150 μg/l for 24h and detected the levels of NF-κB p65 in the cytosolic and nuclear fractions by immunoblotting. We found that the nuclear translocation of NF-κB p65 subunit increased in response to rhHMGB1 stimulation (Figure 3A). Then, we measured the expression of NF-κB p65 when osteoblasts were pretreated with TLR2- or TLR4-siRNA for 24 h, followed by the addition of rhHMGB1 (150 μg/l) to the culture medium for 24 h. As shown in Figure 3B, pretreatment with TLR2- or TLR4-siRNA decreased nuclear NF-κB p65 subunit levels (*P*<0.05), which indicated that rhHMGB1 could induce NF-κB activation via TLR2 or TLR4 in osteoblasts.

### Involvement of TLR2 and TLR4 in HMGB1-mediated osteoblast migration

**Figure 4:**
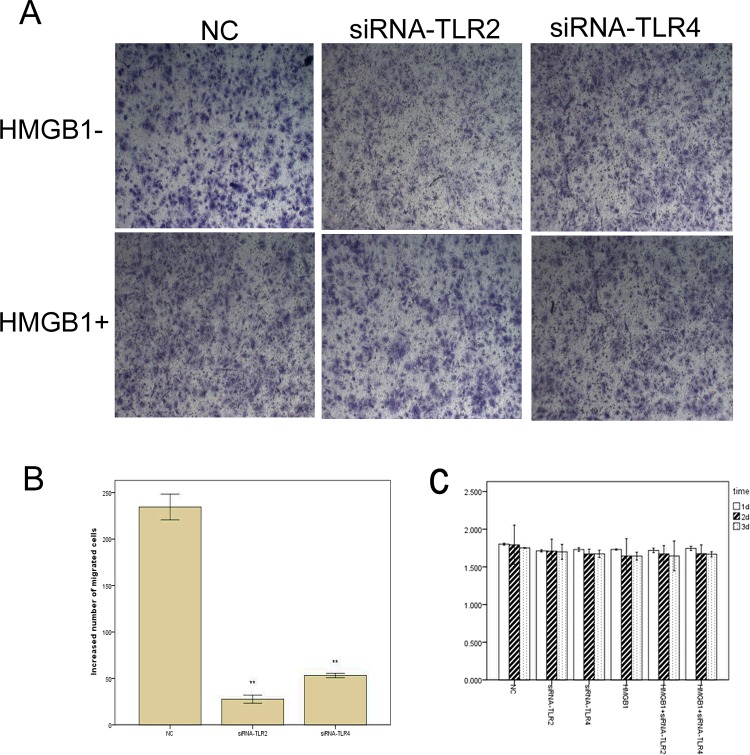
TLR2- or TLR4-siRNA attenuated the mobility of osteoblasts *in vitro* (**A**) Osteoblasts were seeded into the upper chamber of the transwell, treated with rhHMGB1, TLR2-siRNA or TLR4-siRNA, and allowed to invade materiel for 5 h. Invasive cells that migrated through the basal membrane to the lower surface were stained with crystal violet dye, which were photographed at 10× magnification. (**B**) Migrated cells were counted under a light microscope in four randomly selected microscopic fields. One-way ANOVA was performed to determine statistical significance. The increased number of migrated cells was markedly reduced when the cells were pretreated by TLR2- or TLR4-siRNA (**P<0.01). (**C**) Osteoblasts were incubated with TLR2- or TLR4-siRNA or rhHMGB1 for 24–72 h, and the MTT assay showed that knockdown of TLR2 or TLR4 had no significant effect on cell viability.

First, to investigate whether TLR2 or TLR4 signalling is involved in HMGB1-induced osteoblast migration, osteoblasts pretreated with TLR2- or TLR4-siRNA were stimulated with rhHMGB1 and subsequently used in a transwell assay to test for effects on migration. The migration of osteoblasts stimulated with rhHMGB1 (150 μg/l) was enhanced by 2-fold compared with those unstimulated (*P*<0.05). Additionally, after pretreatment with TLR2- or TLR4-siRNA, the increased number of migrated cells was markedly reduced (*P*<0.01; Figures 4A and 4B). Second, pretreated osteoblasts were subjected to the MTT assay to examine proliferation. We found that knockdown of TLR2 or TLR4 did not inhibit osteoblast proliferation compared with those cells stimulated only with HMGB1 (Figure 4C). In accord with our previous findings, these data suggest that TLR2- or TLR4-dependent NF-κB signalling pathways are involved in HMGB1-induced osteoblast migration.

## DISCUSSION

Extracellular HMGB1 has recently been reported to be a multifunctional cytokine that may mediate the processes of embryonic development, vasculogenesis [29], inflammation [10,30], tumorigenesis, skeletal muscle ischemia/reperfusion and lung injury [31]. Cell migration generally begins in response to extracellular stimuli, such as cytokines, extracellular matrix or surrounding cells. Osteoblast migration is responsible for embryo skeletal development and bone tissue repair [32]. Cytokines released by injured bone cells can directly or indirectly act on osteoblasts. The key event during skeletal development is that epiphysis is progressively replaced by bone. Previous reports have shown that HMGB1 can accumulate in the chondrocyte hypertrophic zone in growth plate during embryonic bone development [23]. Additionally, the release of HMGB1 may occur through cell injury or death. A previous report indicates that osteoblasts express all of the known receptors for HMGB1 [22], which supports the hypothesis that higher local concentrations of HMGB1 at sites of tissue damage or endochondral ossification centres might induce osteoblast migration and thereby regulate bone repair or skeletal development.

In the present study, we found that HMGB1 can promote osteoblast migration. The migration rate of osteoblasts increases 2.3-fold after HMGB1 treatment. Previous reports showed that HMGB1 could act as a chemoattractant for mesenchymal stem cells (MSCs). This finding suggests that its persistence in an ossification centre may be required to recruit osteoblasts and MSCs into the site. Additionally, HMGB1 is also an ‘alarmin’ that can be released by dead and dying cells. The passive release of HMGB1 from dying cells is a hallmark of necrotic damage. Meanwhile, many reports have shown that HMGB1 is a potent bone-resorption signal [33,34], which suggests that HMGB1 might play an important role in bone restoration through reducing the migration of osteoclasts and osteoblasts. These data are similar to those of recently published reports in which HMGB1 was shown to induce endothelial cell migration and sprouting, as well as neovasculariza-tion [35].

Extracellular HMGB1 can induce complex signalling cascades by binding to its receptors, which include RAGE, TLR2 and TLR4. Many of these cascades are mediated by well-conserved pathways, such as the MAP kinase, NF-κB and AP-1 transcriptional responses [36,37]. Several studies have associated the local expression of HMGB1 and TLRs with tissue damage and inflammation. HMGB1 can also acts as a regenerative cytokine. Both HMGB1 and TLR4 promote skeletal muscle recovery after ischemia [12]. A recent report demonstrated that its delivery in a mouse model of myocardial infarction could induce myocardial regeneration by activating ‘resident’ cardiac stem cells [38]. In our cellular system, the expression level of TLR2 was higher than that of TLR4. Introduction of specific siRNA constructs could effectively inhibit the expression of the receptor. Our findings suggest that knockdown of TLR2 or TLR4 could significantly inhibit osteoblast migration and NF-κB p65 subunit nuclear translocation when stimulated by HMGB1. Meanwhile, osteoblast proliferation was not significantly inhibited by HMGB1. These data showed that HMGB1 could promote the migration of osteoblasts through TLR2/4 and NF-κB and did not cause any cytotoxic effects. Collectively, our findings suggest that interactions between HMGB1, TLRs and the classical NF-κB pathway are critical to osteoblast migration. Nevertheless, TLR2/4 knockdown by siRNA could not completely abolish osteoblast migration mediated by HMGB1, which could be explained by its interaction with other membrane receptors. Further studies will be required to address these possibilities.

In these bone-related inflammatory disorders, such as rheumatoid arthritis, amount of HMGB1 were released to the extracellular milieu. A previous report showed that HMGB1 exerts a synergistic effect with RANKL to regulate osteoclastogenesis in inflammatory disorders [33]. Therefore, how to prevent the pathological actions of HMGB1 and promote their repair responses may represent a therapeutic strategy for treating diseases in which regeneration is impaired.

Together, our results demonstrate that HMGB1 promotes the migration of osteoblasts by TLR2/4-dependent signalling pathways that drive the activation of NF-κB, which indicates a significant functional role for HMGB1 in skeletal development and bone restoration. To date, signals involved in the extracellular release of HMGB1 are not understood. In monocytes, HMGB1 accumulates in secretory lysosomes that can undergo exocytosis [39]. Whether a similar mechanism occurs in osteoblasts remains unknown. Further studies will be required to assess these questions.

## Abbreviations

α-MEM: alpha minimal essential media
DMEM: Dulbecco's modified eagle media
HRP: horseradish peroxidase
MSC: mesenchymal stem cell
NC-siRNA: negative control siRNA
NF-κB: nuclear factor-kappa B
RAGE: receptor for advanced glycation end products
rhHMGB1: recombinant human high mobility group box 1 protein
TLR: Toll-like receptor
Wnt: wingless and int
